# Reply: Screening for colorectal cancer with immunological FOBT

**DOI:** 10.1038/sj.bjc.6601800

**Published:** 2004-04-13

**Authors:** Hiroshi Saito, M Nakajima

**Affiliations:** 1 Research Center for Cancer Prevention and Screening, National Cancer Center, 5-1-1 Tsukiji, Chuou-ku, Tokyo, 104-0045, Japan; 2 Hirosaki University School of Medicine, 5 Zaifu-cho, Hirosaki 036, Japan

**Sir**,

Dr Otto claimed potential improvement in the sensitivity of immunochemical faecal occult blood testing (IFOBT) by combining faecal albumin detection with IFOBT (Otto and Dobrossy, 2004). As was indicated in their letter, the American Cancer Society special advisory group stated that IFOBT is the only exception to the conclusion that there is insufficient evidence to recommend any emerging technologies as a routine screening test for colorectal cancer (Levin *et al*, 2003). Although this statement needs to be further explained and thus will be discussed later, the point is that IFOBT has been certified as an efficacious tool in terms of evidence. Accordingly, the test used by Dr Otto might be of importance if it could demonstrate even better sensitivity and specificity than those seen with IFOBT, such as the immunochemical haemagglutination test (Saito *et al*, 1984; Saito and Yoshida, 1996).

Since parallel testing generally enhances sensitivity of single testing, it is possible that the addition of faecal albumin detection to IFOBT might improve sensitivity as compared to the detection of faecal haemoglobin alone. The data described in Dr Otto's letter, however, failed to demonstrate the sensitivity of the test in the population, nor did it compare its sensitivity with that of other IFOBT. Although performance characteristics of many available IFOBTs appear generally to be high, difference in sensitivity and specificity among these immunochemical FOBTs has been reported (Saito and Yoshida, 1996). The method employed by Dr Otto and a colleague is a combination test involving immunochemical detection of faecal haemoglobin and albumin, and the sensitivity of their faecal haemoglobin test is unclear. Therefore, the test needs to be compared with IFOBT in the same population for their conclusion to be justified.

Degradation of haemoglobin during transit through the gastrointestinal tract is a well-known phenomenon. Haemoglobin from lesions proximal to the colon is generally believed to lose its antigenicity, a fact that confers an advantage to IFOBT, because a positive test is specific for bleeding from lesions in the colon (Young *et al*, 2002). During transit within the colon, loss of antigenicity also occurs depending on the duration of exposure of haemoglobin to microbial flora. Thus, in theory, colorectal cancer in proximal colonic sites such as the caecum might be more likely to be missed by FOBT. However, it was reported that the sensitivity of IFOBT does not differ between proximal and distal cancers (St John *et al*, 1993). This could be because the effect of bacterial degradation of immunoreactive haemoglobin during transit through the colon might be offset by the high sensitivity of the IFOBT and the fact that proximal lesions bleed more heavily (Macrae and St John, 1982; St John *et al*, 1992). As a result, there has been no firm evidence that sensitivity of FOBT is reduced due to degradation of haemoglobin during transit in the colon.

False-negative testing after degradation of haemoglobin is important in terms of storage of specimens; that is, haemoglobin loses its antigenicity with duration of storage depending on the temperature (Saito *et al*, 1992). Thus, seeking a marker other than haemoglobin in faeces is a reasonable approach. From this point of view, we measured plasma proteins (immunoglobulin, albumin, transferrin, *α*_1_-acid glycoprotein and complements) in faeces, but could not find any that remained significantly more stable than haemoglobin.

Although the IFOBT is treated as an emerging technology in the field of population screening (Levin *et al*, 2003), this is not the case for the immunochemical haemagglutination test, which we developed (Saito *et al*, 1984; Saito, 1996). This has already been used as a population screening test and a reduction in risk of dying from colorectal cancer after screening with this test has been consistently suggested by several studies, although these were observational (Saito *et al*, 1995, 2000; Saito, 1996; Zappa *et al*, 1997). In our most recent study, reduction in risk of developing advanced colorectal cancer has additionally been suggested (Nakajima *et al*, 2003). Furthermore, it was demonstrated that sensitivity was higher for this test than for the Haemoccult test (St John *et al*, 1993; Allison *et al*, 1996; Saito and Yoshida, 1996). Taking into consideration the fact that this test does not require dietary restriction, it undoubtedly has the advantage over the Haemoccult, for which effectiveness has been established. Incorporating IFOBT, including the immunochemical haemagglutination test, the Japanese national screening programme has been run using two-day IFOBT since 1992 with more than 5 million screenees annually.

It is now clearly recognised in many countries, in which the burden of colorectal cancer is serious, that screening should be conducted aimed at reducing the mortality of this disease. Although sensitivity of the IFOBT is not total, it should be the preferred option in terms of evidence, among the modalities available as population screening tools at the present time.
